# In-Vitro Phenotypic Response of Human Osteoblasts to Different Degrees of Titanium Surface Roughness

**DOI:** 10.3390/dj10080140

**Published:** 2022-07-29

**Authors:** Muataz A. Osman, Rasha A. Alamoush, Evgeny Kushnerev, Kevin G. Seymour, Susan Shawcross, Julian M. Yates

**Affiliations:** 1 Division of Dentistry, School of Medical Sciences, Coupland 3 Building, University of Manchester, Oxford Road, Manchester M13 9PL, UK; evgeny.kushnerev@manchester.ac.uk (E.K.); kevin.seymour@manchester.ac.uk (K.G.S.); julian.yates@manchester.ac.uk (J.M.Y.); 2 Periodontology Department, Faculty of Dentistry, The University of Benghazi, Benghazi, Libya; 3 Restorative Department, Faculty of Dentistry, Libyan International Medical University, Benghazi, Libya; 4 Blond McIndoe Laboratories, Division of Cell Matrix Biology & Regenerative Medicine, Faculty of Biology, Medicine & Health, The University of Manchester, 3.106 Stopford Building, Oxford Road, Manchester M13 9PT, UK; sue.shawcross@manchester.ac.uk; 5 Prosthodontic Department, School of Dentistry, University of Jordan, Amman 11942, Jordan; rasha.alamoush@postgrad.manchester.ac.uk

**Keywords:** surface roughness, titanium, titanium surface roughness, human osteoblasts, cell proliferation, cytotoxicity

## Abstract

Objectives: This study aimed to investigate human osteoblast (HOB) responses towards different degrees of titanium (Ti) implant surface roughness. Methods: Four degrees of Ti surface roughness were investigated on a micrometer roughness scale: smooth (S: 0.08–0.1 µm), minimally rough (MM: 0.3–0.5 µm), moderately rough (MR: 1.2–1.4 µm), and rough (R: 3.3–3.7 µm). HOB cells were cultured, expanded, and maintained according to the supplier’s protocol. Cell proliferation and cytotoxicity were assessed at day 1, 3, 5, and 10 using alamarBlue and lactate dehydrogenase colorimetric assays. Data were analyzed with one-way ANOVA, two-way ANOVA, and Tukey’s post hoc test (*p* = 0.05 for all tests). Results: There was no significant difference in the cell proliferation or cytotoxicity of the HOB cells in contact with the different degrees of Ti surface roughness. There was, however, a significant time effect on cell proliferation (*p* < 0.0001) with different exposure durations for each roughness degree. Furthermore, a positive correlation (non-significant) between proliferation and cytotoxicity was observed for all investigated degrees of surface roughness. Conclusion: All investigated roughness degrees showed comparable HOB proliferation, with the MR surface presenting the highest percentage, followed by the R, MM, ad S, surfaces, respectively. The S surface showed the highest cytotoxic effect on HOBs; however, it did not reach the cytotoxic level suggested by the ISO for any medical device to be considered cytotoxic.

## 1. Introduction

Since the time of the pioneering research of Brånemark, Albrektsson, Zarb, and others in the field of osseointegration in the 1950s–1980s [1,2,3,4], the material of choice when making dental and orthopedic implants is still Ti and its alloys, even though increasing attention has been paid to alternative materials in recent years, including zirconia (Zr), polyether ether ketone (PEEK), and others [5].

Ti implants are still considered the gold standard for oral implantology, primarily due to their excellent biocompatibility, their mechanical properties, their ability to form an intimate bone–implant contact with living bone through a cement-free connection at the microscopic level, and their corrosion resistance [5,6,7,8]. These characteristics ensure the successful long-term functioning of the attached protheses [9]. An instantaneously forming, passive Ti oxide (TiO_2_) layer on the surface leads to corrosion resistance and enhanced biocompatibility [10,11,12]. Furthermore, Ti is amenable to alterations and changes in physical and chemical properties through adjustments of the surface oxide composition, thickness, and microtopography, thus enabling property enhancement through surface modification. [13].

Biocompatibility is the ability of a material to prevent the development of an immune response and foreign body reaction and/or rejection when introduced to the human body [14]. The initial interaction between any introduced material and the host tissues starts with a rapidly forming, thin zone of protein adsorption and the formation of a connective tissue interface [13]. This interaction is influenced by the physical and chemical properties of the implant, such as surface roughness, structure, composition, and defects, as well as the thickness of the TiO_2_ layer. These properties are also critical for the long-term success and survival rate of any dental implant [13,15].

Biological materials can be classified as: (1) bio-tolerant, characterized by the formation of a thin, fibrous tissue layer; (2) bio-inert, such as Ti, which can undergo direct bone contact under osteo-permissive conditions; and, finally, (3) bio-active, such as calcium phosphate ceramics, which can achieve a high degree of direct contact with the surrounding bone due to the release of free calcium and phosphate ions at the implant–bone interface [16]. The biocompatibility and inertness of Ti and its alloys are related to the presence of the TiO_2_ layer, which interacts favorably with water ions and serum proteins, as well as their enhanced corrosion resistance [17,18].

Several in vitro studies have illustrated that successful osseointegration between bone and Ti dental implants depends on the macro- and micro-surface topography, chemical composition, and surface energy of the implant [19,20,21]. Surface roughness is an important factor that can influence the biological interactions between cells, tissues, and biomaterials [21]. It has been suggested that dental implant and abutment surface topography and roughness degrees may have an impact on peri-implant soft tissue health and bone levels and, consequently, may affect the incidence of biological complications, such as inflammation, infection, bone loss, implant mobility, and, eventually, implant loss [22].

Various physical, chemical, and mechanical methods have been applied to Ti-based biomaterials to produce micrometer surface structures to help stimulate bone–implant contact. The most common surface treatments include machining, milling, acid-etching, grit-blasting, electrochemical methods, and deposition of ions, proteins, and antimicrobial agents [19]. In vivo studies have demonstrated that bone tends to form preferentially on R surfaces, whereas connective tissue forms more favorably on S surfaces [23,24].

Considering the trans-gingival nature of dental implants, forming a number of simultaneous interfaces with the host biological system, it is important to understand the different cells and tissues involved in this process. These interfaces consist of: (1) the implant body–bone interface, (2) the soft tissue interface at the level of the implant neck/platform, and, finally, (3) the soft tissue interface at the junction of the supra-gingival region [5]. Each surface of the dental implant should be optimized to fulfil the different demands of the respective interface. For example, at the implant body level, osteogenic properties are required to optimize bone contact, formation, maturation, and subsequent osseointegration, whilst at the soft tissue interface, gingival attachment with cell-adhesion abilities for fibroblasts and keratinocytes is essential to ensure a tight epithelial seal around the implant neck and its abutment in order to prevent bacterial infiltration and inflammation [25].

After implant placement, several crucial cellular interactions create a strong bone-to-implant connection. It is essential that cells adhere to the implant surface, and the surface roughness of dental implants can have a significant influence on HOB adhesion in the early phase of healing, as well as improving and accelerating the osseointegration process [22]. Other important factors are biocompatibility and resistance to bacterial infiltration and contamination [21]. The bio-inert property of Ti is ensured by the protective layer of TiO_2_ that forms on its surface. This layer prevents the penetration of metal compounds, and calcium and phosphate ions are readily able to adhere to the surface, which is necessary for the formation and maturation of the mineralized bone structure. Since the presence of this layer alone is not sufficient for the biocompatibility of Ti, a suitable surface finish and roughness is required to help create and enhance a strong bone-to-implant connection and improve the long-term success following implant therapy [21,26].

Surface roughness values can either be calculated on a profile (line/2D) or on a surface (area/3D). The profile roughness parameters are Ra, Rq, etc. The area roughness parameters are Sa and Sq, which give more meaningful values (defining the height of the surface topography); hence, they have been employed recently as the parameters of choice to describe surface roughness in implant dentistry [27]. Dental implant surfaces can be classified into four different groups according to their surface roughness: S surfaces with Sa values of less than 0.3 µm, MM surfaces with Sa values of 0.3 to less than 1.0 µm, MR surfaces where Sa values are between 1.0 and 2.0 µm, and, finally, R surfaces where the Sa values are greater than 2.0 µm [28].

Despite the reported clinical benefits and advantages of the currently available surface-modified implants, some scientific reports suggest that surface roughness may play a key role in the accelerated development of the bone–implant connection in the short term and also of peri-implant soft and hard tissue damage, which may be either reversible (peri-implant mucositis) or irreversible (peri-implantitis), in the long term [22,29]. This study aimed to investigate human osteoblast (HOB) responses towards various degrees of Ti (Ti6Al-4V) implant surface roughness (created using a simple industrial milling machine) and to determine if this surface roughness influences early-stage HOB proliferation in the same way that other surface treatments do, which in turn may potentially influence bone healing.

The null hypotheses are:

- There is no difference in the proliferation of the HOB cells in contact with Ti discs of various degrees of surface roughness;
- There is no difference in the cytotoxicity of the HOB cells in contact with Ti discs of various degrees of surface roughness and that;
- There is no effect on either the proliferation or cytotoxicity of the HOB cells with different exposure times for each surface roughness degree.

## 2. Materials and Methods

### 2.1. Specimens’ Preparation

**Figure 1:**
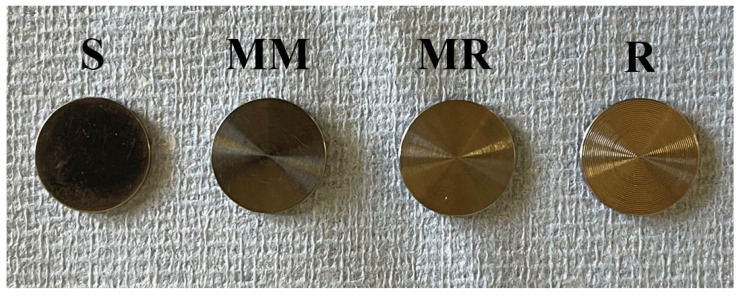
Ti discs with different roughness degrees (S, MM, MR, and R).

Eighty-eight Ti Ti6Al-4V (grade 4 cert:20) discs (Figure 1) were produced and received from a commercial supplier (GC Tech. Europe GmbH, Harkortstr. 2, D-58339 Breckerfeld, Germany), with four different roughness groups: S (*n* = 22), MM (*n* = 22), MR (*n* = 22), and R (*n* = 22) R. The discs’ dimensions were: 12 mm in diameter and 1 mm thickness modified on both sides. Different surface roughness degrees were produced using a milling machine with a milling bur that could be adjusted between 0.002 and 0.05 mm. Discs were then washed using industrial ultrasound equipment (ATU Ultrasonidos^®^, Brussels, Belgium) in a soapy solution at 60 °C to eliminate traces of oil and/or other residue derived from the machining process without damaging the surfaces. Discs were then dried in a forced convection oven for 1 h (JP Selecta^®^, Brussels, Belgium) and then autoclaved under steam pressure at 121 °C for 20 min according to the supplier’s recommendations.

### 2.2. Surface Roughness Measurements

The surface roughness of the Ti discs was measured using a 3D non-contact high-resolution contour GT-K optical surface profiler (Veeco ContourGT™, Tuscon, AZ, USA). To perform the measurement, the vertical scanning interferometry (VSI) mode was selected. The 50× objective lens, which provides an area of 174.7 (x) and 132 (y) microns, was used. A Gaussian regression filter with a short wavelength cut-off of 25 μm (0.025 mm) was applied before determining the surface roughness parameters. Two samples from each group of investigated surfaces were measured at three random points on each side (six points in total per disc). Five different surface roughness parameters were measured as per the data from the profilometer software: mean surface roughness (Sa), maximum valley depth from mean plane (Sv), skewness (Ssk), developed surface area (Sdr %), and density of summits (Sds). However, the Sa value, “the arithmetic mean of values above and below a mean plane”, was used to represent the Ti surface roughness, as recommended by dental implant research methodology and scientific papers [5,30].

### 2.3. Surface Morphology Analysis

The surfaces (*n* = 2) of the discs of each group were also examined using a scanning electron microscope (SEM) (Quanta™ FEG 250 SEM, Edificio I+D-Campus Río Ebro C/ Mariano Esquillor s/n 50018 Zaragoza, Spain). The images were obtained using the following parameters: 500 µm magnification, accelerating voltage of 20 kV, spot size of 3.0, and working distance (WD) of 7.6–7.9 mm.

The use of the SEM in this study allowed the imaging of the surface roughness degree of the Ti discs and visual observation of the differences between the different investigated groups.

### 2.4. HOB Cell Culture Preparation

Primary human osteoblasts (HOBs) (lot no.: 445Z009.2) were obtained from PromoCell (Heidelberg, Germany), which were derived from human hipbone (femur of a fit and healthy 61 year old female) biopsies. The cells were cultured according to PromoCell guidelines, and the procedure described by Alamoush et al. [31]. Cells were cultured in osteoblast growth medium (OGM) and mixed with SupplementMix supplied by the same company. Culture medium was free of antibiotics as per the manufacturer’s recommendations. The cells were processed at regular periods based on their growth characteristics and the supplier’s protocol. Cells were incubated at 37 °C and 5% CO_2_ (Panasonic CO_2_ Incubator, MCO-170AIC, Panasonic Healthcare Co. Ltd., Tokyo, Japan). Ti discs were placed in 24-well plates in 500 µL OGM and incubated for 24 h before seeding the cells. When they reached 70–90% confluence, cells were detached using 0.25% Trypsin-EDTA (Gibco™, Life Technologies, Inc., Burlington, ON, Canada). Cells were then counted using a Millipore Scepter counter (Merck Millipore, Watford, UK) and 5 × 10^4^ cells were seeded on each disc in a 24-well culture plate (Corning Costar Ultra-Low Attachment Multi-Well Plates (Corning Inc., Corning, NY, USA)) in 500 µL of complete growth medium.

All experiments were performed using suitable controls with biological and instrumental triplicates and replicated at least three times.

### 2.5. Cell Viability

Cellular viability of 100% was attributed to control wells, where cells were cultured with no Ti discs (positive growth control). Cellular viability was quantified via a colorimetric assay using an Invitrogen alamarBlue™ Cell Viability Reagent (DAL1100, lot: 2120063 (Life Technologies Corporation, Thermo Fisher Scientific, Waltham, MA, USA)). Cell viability was measured at days 1, 3, 5, and 10 of cell growth. HOB cells were exposed to alamarBlue™ (1:10, reagent:OGM) for 1 h at 37 °C at each time point. Then, 100 µL of supernatant was transferred into a 96-well plate in triplicates for analysis at each time point. The 96-well plate (Corning Costar Ultra-Low Attachment Multi-Well Plates (Corning Inc., Corning, NY, USA)) was read with a UVM 340 microplate reader at 570 nm and 600 nm (ASYS, Scientific Laboratory Supplies). Cell viability was calculated according to the following equation [32]:

$$\mathrm{Cell}\;\mathrm{viability}\;\%=\frac{A570\;–\;\left(A600\;x\;R_{\circ }\right)\;\mathrm{for}\;\mathrm{test}\;\mathrm{well}\;}{A570-\left(A600\;x\;R_{\circ }\right)\;\mathrm{positive}\;\mathrm{growth}\;\mathrm{control}\;}\;\times \;100$$

where A570 and A600 are the absorbance at 570 and 600 nm, respectively, and $R_{\circ }$ is the correction factor calculated from A570/A600 for the positive growth control.

### 2.6. Cytotoxicity

Cell cytotoxicity was evaluated using a CyQUANT™ LDH Cytotoxicity Assay kit (C20301, Thermo Fisher Scientific, Waltham, MA, USA). Cytotoxicity for HOBs was measured at days 1, 3, 5, and 10. At each of the four time points, as per the company’s protocol, 50 µL of lysis buffer (containing membranolytic particles) was added to the specific time-point wells (maximum LDH release, high control, HC), 50 µL of sterile-filtered BioReagent water (SIGMA-ALDRICH^R^, Life Science, Glasgow, UK) was added to the low-control wells (spontaneous LDH release), and the plates were incubated at 37 °C in 5% CO_2_ for 45 min. The cytotoxicity was then measured using 50 µL of the supernatant and 50 µL of LDH cell reaction solution, then incubated for 30 min at room temperature in the dark. The reaction was stopped using 50 µL of the LDH kit stop solution. The 96-well plate (Corning Costar Ultra-Low Attachment Multi-Well plates (Corning Inc., Corning, NY, USA)) was read with a UVM 340 microplate reader at 490 nm subtracted from 680 nm (ASYS, Scientific Laboratory Supplies), and cytotoxicity was calculated according to the following equation [33]:

$$\mathrm{Cytotoxicity}\;\%=\frac{\mathrm{Specimen}-\mathrm{treated}\;\mathrm{LDH}\;\mathrm{activity}\;-\;\mathrm{Spontaneous}\;\mathrm{LDH}\;\mathrm{activity}\;}{\mathrm{Maximum}\;\mathrm{LDH}\;\mathrm{activity}\;-\;\mathrm{Spontaneous}\;\mathrm{LDH}\;\mathrm{activity}\;}\;\times \;100$$

where the specimen-treated LDH activity is the LDH amount expressed by cells cultured with Ti discs, the maximum LDH activity is the LDH amount expressed by cells treated with lysis buffer, and the spontaneous LDH activity is the LDH amount expressed by cells treated with sterile water.

### 2.7. Statistical Analysis

Data were analyzed (GraphPad Prism, version 9.1.2 (226)) in statistical software and found to be normally distributed (Shapiro–Wilk’s test). Two-way ANOVA was performed for the roughness effect, time effect, and the interaction between different surface roughness degrees, followed by one-way ANOVA and Tukey’s multiple comparisons to compare cell viability and cytotoxicity for different roughness degrees at each time point (significant *p* value = 0.05 for all tests). 

## 3. Results

### 3.1. Surface Roughness

**Figure 2:**
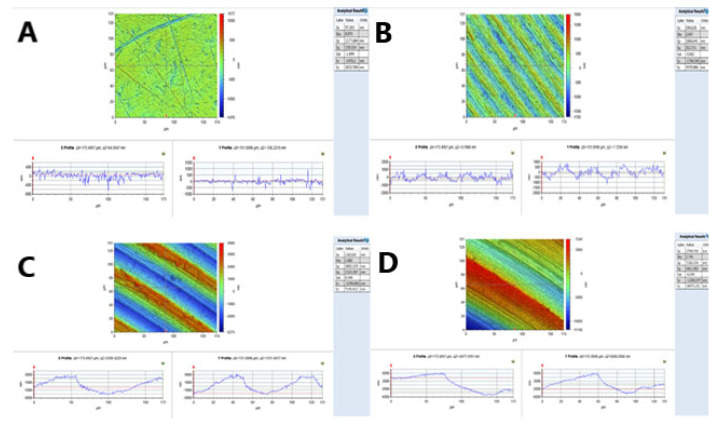
Profilometer surface roughness assessment and analysis: (**A**) Ti S surface, (**B**) Ti MM surface, (**C**) Ti MR surface, (**D**) Ti R surface.

Sa values (mean–standard deviation (SD)) obtained for the S (0.11 µm), MM (0.39 µm), MR (1.33 µm), and R (3.34 µm) (Figure 2) surfaces are presented in (Table 1) below, as well as the maximum and minimum Sa values for three randomly selected points on each disc surface.

### 3.2. Surface Morphology Analysis

**Figure 3:**
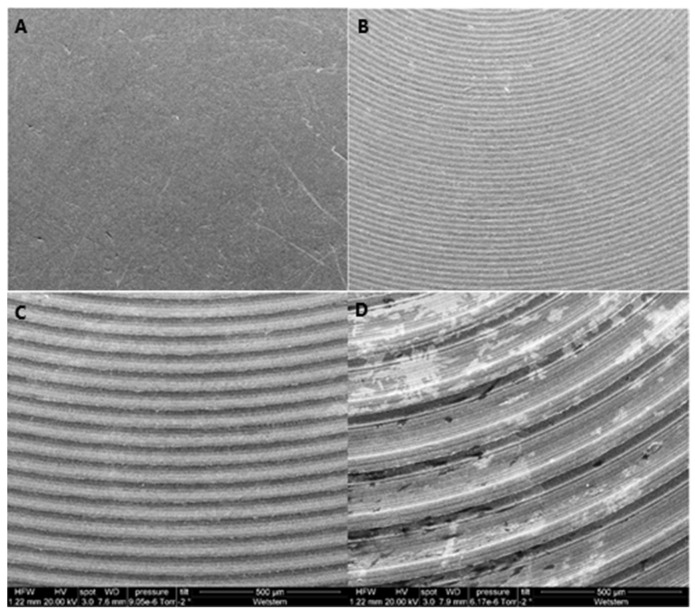
SEM images: (**A**) Ti S surface, (**B**) Ti MM surface, (**C**) Ti MR surface, (**D**) Ti R surface.

The qualitative analysis (using images) of the different roughness groups studied is illustrated in Figure 3.

As demonstrated by the pictures taken with the SEM (Figure 3), the differences in surface roughness for the four samples could be clearly seen. The S surface sample showed a regular, slightly granular surface, with some surface imperfections visible in the top left-hand side of the image (A). The other three samples showed concentric lines cut by the machining process, leaving peak and trough patterns. These increased in depth and width as the surface roughness changed from MM (B) to MR (C) to R (D). In addition, there appeared to be two levels of machining in each sample, such that there seemed to be a further trough cut into each peak. This was not so visible in the minimally rough and moderately rough samples but was clearly visible in the rough surface.

### 3.3. Cell Viability

There were no statistically significant differences in the proliferation (P%) of HOBs in contact with the four different degrees of roughness of the Ti discs at each time point tested (days 1, 3, 5, and 10). The highest proliferation on day 1 was expressed by the cells in contact with the S surface discs (85.36%), followed by the MM surface (84.99%), and then the MR surface (81.54%), and the lowest proliferation was expressed by the cells in contact with the R surface (79.49%). However, although differences in P% were observed, none of the differences between tested surfaces were significant.

**Figure 4:**
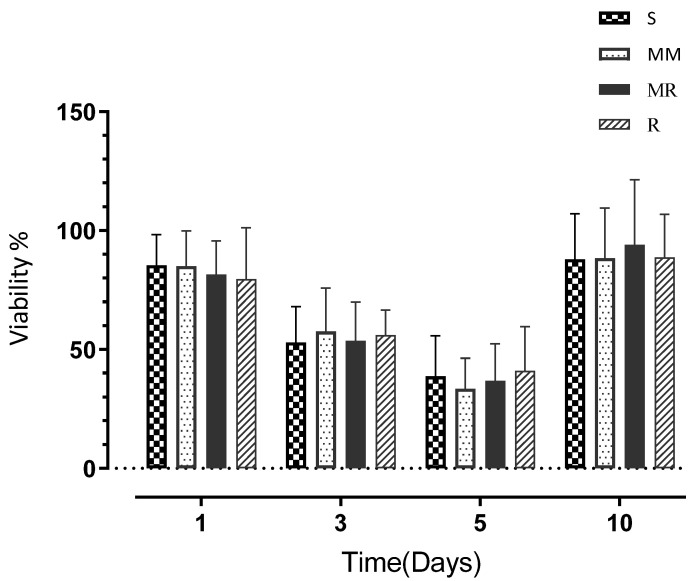
A bar chart demonstrating the M P% values at days 1, 3, 5, and 10 for HOBs. Error bars represent the SD.

At day 3, the P% dropped by almost 25% compared to day 1 values for all the tested samples (Figure 4), and this decline in the proliferation pattern continued until day 5, at which point the lowest P% was expressed by the MM surface (33.46%). The S and MR surfaces showed P% values of 38.66% and 36.69, respectively, and the maximum P% was expressed by the R surface (41.10%). At day 10, the cells expressed P% values higher than at day 1, and the highest P% was expressed by cells in contact with the MR surface (93.96%), followed by the R surface (88.63%) and then the MM surface (88.32%), with the S surface demonstrating the lowest P% at 87.90% (Table 2). At day 10, all Ti surface roughness degrees illustrated comparable results in terms of HOB P%, with the MR surface showing the highest P% (Figure 4).

The two-way ANOVA analysis showed a significant time effect on cell proliferation (*p* < 0.0001) but no significant effects on the different surface roughness degrees and no significant interactions.

### 3.4. Cell Cytotoxicity

**Figure 5:**
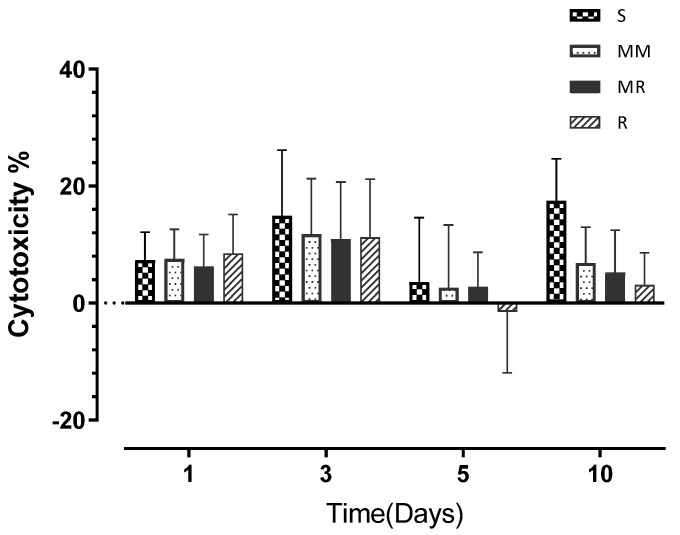
A bar chart illustrating the M cytotoxicity percentage values at days 1, 3, 5, and 10 for HOBs. Error bars represent the SD.

HBO cytotoxicity was generally the highest on day 3, but without statistically significant differences between the investigated roughness degrees (Table 3), and it demonstrated the following order from highest to lowest: S > MM > R > MR with cytotoxicity percentages of 14.90% > 11.74% > 11.25% > 10.94%, respectively. The highest cytotoxicity percentage throughout the whole experiment was 17.45% at day 10, exhibited by the S surface (Figure 5). In general, cytotoxicity was lower than 10% on day 1, increased on day 3 to a maximum of 14.90% (demonstrated by the S surface), dropped to the lowest values for all tested surfaces on day 5, and slightly increased on day 10.

The two-way ANOVA analysis highlighted significant time (*p* < 0.0001) and material (*p* < 0.03) effects on cell cytotoxicity but no significant interactions.

**Figure 6:**
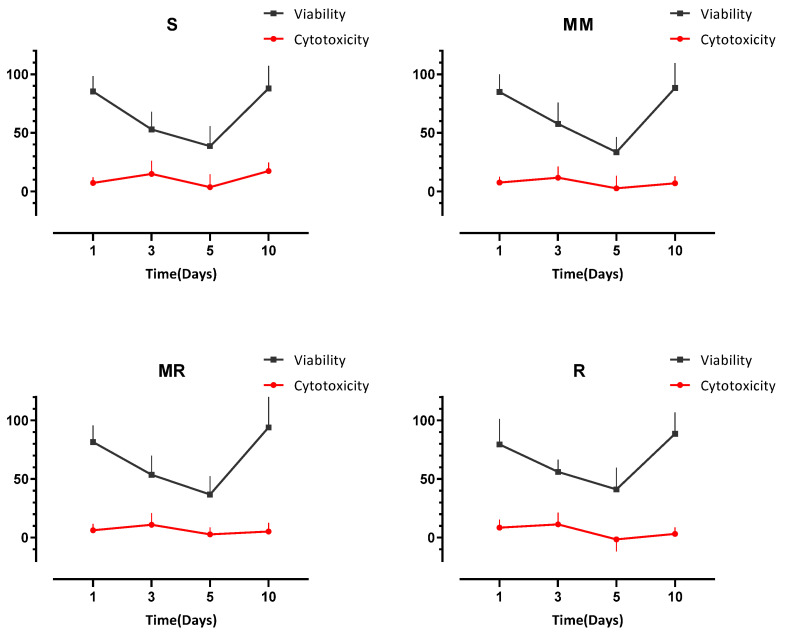
Line plots showing positive but non-significant correlations between proliferation and cytotoxicity for all surfaces from day 3 to day 10 and a non-significant negative correlation at day 1.

Both assays were compared in terms of the degree of surface roughness at each time point (Figure 6). Non-significant positive correlations between viability and cytotoxicity were found for all surface roughness degrees investigated from day 3 to day 10 and a non-significant negative correlation at day 1.

## 4. Discussion

After the insertion of dental implants, the jawbone interacts with the implanted surface and the characteristics of that surface affect the synthesis and release of the local factors, such as inflammatory mediators, cytokines, and growth factors, produced by the surrounding cells and tissues that adhere to the implant surface, including mesenchymal cells and osteoblasts. These local factors affect the process of bone formation and maturation and wound healing and eventually influence titanium biocompatibility and osseointegration/contact with bone [34].

The surface structure and roughness can significantly affect the proliferation and protein synthesis of the HOBs that are cultured on metal substrates [34], and this may have an effect on bony growth, especially in the early phase of healing.

Numerous techniques have been developed during the last 50 years aiming to improve bone-to-implant contact and accelerate osseointegration from a physical and chemical perspective [35]. The earliest osseointegrated implant surfaces were produced with industrial machining techniques, which led to minimally to moderately rough surfaces with residual intermittent microgrooves. Despite the clinical success of these machined surfaces, further methods have been developed to improve microtopography and surface roughness and achieve a larger surface area—which leads to better bone-to-implant contact and faster osseointegration—using either additive or subtractive methods. These methods include, but are not limited to, Ti plasma spraying, acid-etching, grit-blasting, anodization, and laser, antimicrobial, and growth-factor coating [36]. However, many of these developments have been driven by clinical observations and not quantified at a cellular level through “in vitro” investigations.

In the present study, four different degrees of Ti surface roughness obtained with the same preparation technique (industrial machining) were investigated in terms of their influence on HOB proliferation and cytotoxicity. The present study found no statistically significant differences in HOB cell proliferation between all the investigated degrees of roughness; thus, the first null hypothesis was accepted. However, the two-way ANOVA analysis highlighted a significant time effect on cell proliferation (*p* < 0.0001).

In term of cytotoxicity, there were no statistically significant differences in the cytotoxic effects between all the tested surfaces when HOB cells were exposed to them. However, on day 10, the S surface showed the highest percentage of HBO cytotoxicity across the whole experiment (17.45%)—more than double the other surfaces’ figures—and the two-way ANOVA analysis revealed significant time (*p* < 0.0001) and surface (*p* < 0.03) effects on cell cytotoxicity. However, no significant interactions were found. Additionally, the 30% cut-off percentage for cytotoxicity was not reached by any of the surfaces at any time point, which implies that all surfaces were biocompatible and non-toxic. These results mean that the second null hypothesis was partially accepted. Furthermore, according to the analysis of our results, there were also significant time effects on both cell proliferation (*p* < 0.0001) and cytotoxicity (*p* < 0.0001); consequently, the third hypothesis was rejected.

Different degrees of surface roughness may result in distinct effects on different living tissues and cells [37]. On a 3D scale, smooth surfaces have been defined as having an average height deviation (Sa) of <0.5 µm, minimally rough surfaces an Sa of 0.5–1 µm, moderately rough surfaces an Sa of 1–2 µm, and rough surfaces an Sa of > 2 µm, which were the values that were used in this study [38,39]. Moderately rough and rough surfaces were associated with stronger bone responses; that is, an enhanced proliferation percentage and fewer cytotoxic effects than the smooth and minimally rough surfaces noticed in this study. These results are in line with several other in vitro and in vivo studies, with moderately rough surfaces having the optimal range of roughness for osseointegration [40,41].

At a cellular level, although it was not investigated in this study, moderately rough surfaces may be better for cell attachment, while rough surfaces leave larger distances between the peaks of the surface, such that bone cells perceive them as flat, causing excessive cell flattening and compromising their attachment and nutrition, thus affecting attachment and proliferation [27].

The impact of macro-roughness on dental implants is primarily mechanical: the surface irregularities mechanically strengthen the implant stability within the jawbone, but they are too large to be influential on cells [42]. On the other hand, micro- and nano-roughness appear to influence osteogenesis and cellular behavior through alterations in mesenchymal stem cell biological functions [37].

The surface chemistry, energy, wettability, and mechanical properties of the commercially available implants might also be different compared to the Ti surfaces used in this experiment [43]; however, these properties were not of interest in this study, as it was purely the biocompatibility of different degrees of surface roughness in the exact same material under static biological conditions, and the quantification of the effects on HBOs, that were being investigated.

As has been reported in many studies [43], an increase in surface roughness leads to an increase in the total surface area of implant surfaces. This means a larger area for the attachment of cells, as well as for the production of important and relevant proteins, inflammatory mediators, and growth factors. This was the case in this experiment, where the MR and R surfaces exhibited greater proliferation and much lower cytotoxicity than the S and MM surfaces. These findings are consistent with previous studies that also reported that increasing the roughness of Ti surfaces elicited enhanced levels of bone cell proliferation in vitro [44,45,46]. In contrast, several authors observed greater cell proliferation on Ti surfaces as roughness decreased [47,48,49,50].

In the present experiment, cell proliferation decreased at days 3 and 5 and then dramatically increased at day 10. In part, this drop in cell proliferation could have been due to the short time interval between both evaluations (2 days), which technically would have allowed less time for the cells to adapt to the new environment and proliferate; however, toward day 10, when the cells had more time to adapt, the proliferation level increased. This decrease in cell proliferation does not necessarily imply that cells are dying, as they might be reacting to the surface through differentiation rather than proliferation, and increased proliferation might be a reaction to environmental stimuli [31,51,52].

The effect of surface roughness on HOB cell behavior and metabolic activity has been shown in previous in vitro and in vivo studies [53,54]. Further findings were reported by Deligianni et al. after investigating the influence of three different degrees of roughness in hydroxyapatite (HA) surfaces (smooth, machined, and rough), fabricated using grinding papers on HA-coated Ti discs [55]. The roughness of the HA Ti surfaces had no impact on bone marrow cell morphology or ALP activity. Nevertheless, cellular proliferation after 14 days was higher in coarser HA compared to smoother surfaces [55], and the same results was observed by Kunzler et al., who reported a significant increase in the number and the proliferation rate of rat osteoblasts after increasing the surface roughness gradients of high-purity aluminum [56]. The increased proliferation results for rougher surfaces are comparable to the results from our study with HOB after 10 days in vitro with machine-created roughness. A period of 10 days may be too short to detect significant differences in the proliferation of HOB on Ti surfaces; however, it does indicate that early populations of HOBs on Ti surfaces may be enhanced by increased roughness.

All the investigated degrees of roughness showed comparable cytotoxicity levels, with the S surface showing the highest values at day 10 with 17.45%; however, none of the values exceeded the ISO standard level of cytotoxic material (30%) [57]. Therefore, this implies that all the surfaces investigated may be suitable for in vivo use and that other characteristics may influence short-term healing and osseointegration.

The surface area of each disc was 223 mm^2^ and thus within the range of the surface area of an 11 mm × 4 mm Ø and 13 mm × 4.5 mm Ø dental implant, which is representative of the clinical situation. Additionally, the Ti discs were incubated after cell seeding at 37 °C to simulate oral conditions [57]. Furthermore, the ratio of the surface area of the sample to the medium volume was 3 cm^2^/mL, which is in accordance with the ISO standard for such investigations (ISO 10933, 12) [58].

A final point to highlight is that an HOB primary cell line was used in these investigations rather than immortalized or carcinoma cells, which were used in other studies as it was believed they are more representative of healthy human cells located within the oral environment [59,60] and represent the most clinically comparable and sensitive method to measure low cytotoxicity levels [61].

## 5. Conclusions

In summary, all investigated roughness degrees showed comparable HOB proliferation rates, with the MR surface presenting the highest percentage, followed by the R, MM, and S surfaces, respectively. The S surface showed the highest inhibitory effect on HOB; however, it did not reach the cytotoxic level suggested by the ISO for any medical device to be considered cytotoxic.

Although an R surface can mechanically increase bone-to-implant contact, it can also stimulate inflammatory cytokine production, which may result in bone resorption [62]; thus, increasing roughness could ultimately lead to soft tissue inflammation, infection, alveolar bone resorption, and, eventually, implant failure and loss.

Finally, it is important to highlight that use of a simple milling procedure to create surface roughness can produce similar/comparable biological effects on HOBs as other more expensive and complicated procedures and techniques.

## Figures and Tables

**Table 1:** Sa mean values and SD for S, MM, MR, and R surfaces with the maximum and minimum Sa values for three randomly selected pointes on each side of the discs.

Roughness Degree	Smooth (S)	Minimally Rough (MM)	Moderately Rough (MR)	Rough (R)
Sa value: mean (µm)	0.11 µm (0.01)	0.39 µm (0.09)	1.33 µm (0.02)	3.34 µm (0.06)
Minimum Sa value (µm)	0.18 µm	0.27 µm	1.25 µm	2.4 µm
Maximum Sa value (µm)	0.08 µm	0.5 µm	1.47 µm	3.79 µm

**Table 2:** Mean (M) and standard deviation (SD) values for alamarBlue cell proliferation percentages at days 1, 3, 5, and 10. Non-significant differences (Tukey’s post hoc test (*p* = 0.05)) between investigated roughness degrees at each time point were found.

Surface Roughness	Smooth (S)		Minimally Rough (MM)		Moderately Rough (MR)		Rough (R)	
Time (days)	P%	SD%	P%	SD%	P%	SD%	P%	SD%
Day 1	85.36	(12.87)	84.99	(14.88)	81.54	(14.12)	79.49	(21.72)
Day 3	52.94	(14.99)	57.57	(18.17)	53.61	(16.30)	56.03	(10.49)
Day 5	38.66	(17.08)	33.46	(18.17)	36.69	(15.70)	41.10	(18.46)
Day 10	87.90	(19.19)	88.32	(21.05)	93.96	(27.36)	88.63	(18.26)

**Table 3:** The M and SD values for HOB cell cytotoxicity percentages at days 1, 3, 5, and 10. Non-significant differences (Tukey’s post hoc test (*p* = 0.05)) between the investigated surface roughness degrees at each time point were found.

Surface Roughness	Smooth (S)		Minimally Rough (MM)		Moderately Rough (MR)		Rough (R)	
Time (Days)	Mean%	SD%	Mean%	SD%	Mean%	SD%	Mean%	SD%
Day 1	7.33	(4.77)	7.53	(5.07)	6.24	(5.48)	8.46	(6.68)
Day 3	14.90	(11.23)	11.74	(9.54)	10.94	(9.79)	11.25	(9.95)
Day 5	3.60	(11.01)	2.64	(10.69)	2.78	(5.89)	−1.49	(10.44)
Day 10	17.45	(7.19)	6.84	(6.15)	5.21	(7.25)	3.13	(5.45)

## Data Availability

Please email the corresponding author and the author will provide data according to the University of Manchester data sharing policy.
